# Chromothriptic Translocation t(1;18): A Paradigm of Genomic Complexity in a Child with Normal Intellectual Development and Pyridoxine-Dependent Epilepsy

**DOI:** 10.3390/genes16111334

**Published:** 2025-11-05

**Authors:** Raffaele Falsaperla, Eliana Salvo, Annamaria Sapuppo, Chiara Barberi, Vincenzo Sortino, Gaia Fusto, Roberta Rizzo, Xena Giada Pappalardo, Giovanni Corsello, Martino Ruggieri, Catia Romano, Lucia Saccuzzo, Marco Fichera, Maria Clara Bonaglia

**Affiliations:** 1 Department of Medical Science, Pediatrics, University of Ferrara, 44124 Ferrara, Italy; raffaele.falsaperla@unife.it; 2 Neonatal Intensive Care and Neonatal Accompaniment Unit, Azienda Ospedaliero-Universitaria Policlinico “Rodolico-San Marco”, San Marco Hospital, University of Catania, 95123 Catania, Italy; 3 Laboratory of Cytogenetics, IRCCS E. Medea, 23842 Bosisio Parini, Italy; eliana.salvo@lanostrafamiglia.it; 4 Unit of Pediatrics, Pediatric Emergency Department, Azienda Ospedaliero-Universitaria Policlinico “Rodolico-San Marco”, San Marco Hospital, 95123 Catania, Italy; annamaria.pan@gmail.com (A.S.); sortino.vinci@gmail.com (V.S.); 5 Postgraduate Training Program in Pediatrics, University of Palermo, 90121 Palermo, Italy; chiara.barberi@hotmail.com; 6 Department of Biomedical and Biotechnological Sciences, Medical Genetics, University of Catania, 95123 Catania, Italy; fustogaia@gmail.com (G.F.); rizzo.roberta@studium.unict.it (R.R.); xena.pappalardo@irib.cnr.it (X.G.P.); 7 Department of Health Promotion, Mother and Child Care, Internal Medicine and Medical Specialties “G. D’Alessandro”, University of Palermo, 90121 Palermo, Italy; giovanni.corsello@unipa.it; 8 Unit of Clinical Pediatrics, Unit of Rare Disease AOU “Policlinico”, PO “G. Rodolico”, University of Catania, 95123 Catania, Italy; m.ruggieri@unict.it; 9 Azienda Sanitaria Provinciale (ASP) di Catania, 95124 Catania, Italy; catia.romano@asp.ct.it; 10 Section of Clinical Biochemistry and Medical Genetics, Department of Biomedical and Biotechnological Sciences, Medical Genetics, University of Catania, 95123 Catania, Italy; lrsa@live.it (L.S.); marco.fichera@unict.it (M.F.); 11 Research Unit of Rare Diseases and Neurodevelopmental Disorders, Oasi Research Institute-IRCCS, 94018 Troina, Italy

**Keywords:** 16p11.2 duplication, 18q12.3 deletion, seizures, RIT2, optical genome mapping, whole-genome sequencing

## Abstract

Background: Pyridoxine-dependent epilepsy (PDE) is a rare disorder characterized by seizures resistant to conventional treatments but responsive to pyridoxine therapy. Typically caused by biallelic variants in *ALDH7A1*, *PNPO*, or *PLPBP*, a few patients present a similar clinical phenotype but without confirmed molecular diagnoses. We report a child with a 13-year PDE diagnosis and normal intellectual development, whose seizures recurred after pyridoxine withdrawal but resolved with reintroduction, despite unremarkable whole-exome sequencing results. Methods: Following negative results from WES, optical genome mapping (OGM) and whole-genome sequencing (WGS) were performed to highlight any potential structural variants involving known PDE-associated genes. Results: OGM and WGS revealed a recurrent 16p11.2 BP4-5 duplication, inherited from his healthy father, along with a de novo chromothripsis-type unbalanced t(1;18)(p22.3;q12.3), affecting several genes not currently associated with epilepsy (*RIT2*, *PIK3C3*, *COL24A1*, *LRRC8D*, *DIPK1A*, and *DPYD*), with *RIT2* being a plausible candidate for the neurological phenotype due to its neuron-specific expression along with a likely reshuffling of topologically associating domains (TADs) involving *SYT4*, an epilepsy-candidate gene. Discussion: While the molecular data do not pinpoint a single gene or locus as the cause of seizures in this case, a key aspect of our patient’s phenotype is true pyridoxine dependence, rather than just pyridoxine responsiveness. We propose that the genomic complexity associated with the chromothriptic t(1;18) and the 16p11.2 BP4-5 duplication may create a unique metabolic environment in which pyridoxine-dependent pathways are disrupted through unconventional mechanisms. The preservation of cognitive function in our case has been observed in small groups of PDE patients, especially those diagnosed and treated early. This may indicate a distinct phenotypic subgroup that warrants further genetic investigation.

## 1. Introduction

Pyridoxine-dependent epilepsy (PDE; OMIM#266100) is a rare, autosomal recessive disorder typically characterized by neonatal seizures resistant to antiepileptic drugs (AEDs) but that responds to drug therapy with pyridoxine (PN). Less frequently, later-onset cases appear during childhood [1]. PDE is caused by an inborn error of PN metabolism, mainly due to the biallelic variant of *ALDH7A1*, a gene encoding a key enzyme in lysine metabolism, which is eventually implicated in the activation of Vitamin B6 [2]. Moreover, *PNPO* and *PLPBP* genes, both involved in the homeostasis of the active form of PN, have recently been associated with PDE [1].

So far, several patients responding to PN without a confirmed molecular diagnosis have been reported and were considered cases requiring further genetic investigation [3,4,5]. Here, we report on a child with normal intellectual development and a clinical diagnosis of PDE for 13 years based on sustained responsiveness to PN, despite unremarkable WES results. OGM, PE-WGS, and real-time PCR (RT-PCR) revealed a de novo complex unbalanced translocation t(1;18)(p22.3;q12.3), consistent with a chromothripsis event and a recurrent 16p11.2 duplication BP4-5, inherited from the healthy father. The complexity of this genomic context and the potential to identify novel genotype-phenotype correlations are discussed.

## 2. Materials and Methods

### 2.1. Clinical Presentation

We reported on a 13-year-old boy affected by PDE, born prematurely at 34 weeks of gestation via cesarean section due to maternal hypertransaminasemia gravidarum. He was the first child of non-consanguineous parents from a triplet pregnancy, conceived through assisted reproductive technology, with an unremarkable perinatal course. His development in the first months of life was characterized by an initial psychomotor delay relative to his twin sisters, low reactivity to stimuli, and impaired grasping. At 5–6 months old, episodes of upward gaze deviation and immobilization lasting a few seconds appeared, leading to the initial diagnosis of epileptic encephalopathy. Brain MRI revealed modest frontal CSF space dilation suggestive of benign external hydrocephalus, mild corpus callosum dysgenesis with posterior thinning, and cerebellar vermis inferior lobe hypoplasia. The first video-EEG showed an epileptic encephalopathy pattern with focal and generalized epileptic abnormalities, predominantly in the temporal regions. Antiepileptic drugs (phenobarbital, levetiracetam, and valproic acid) were started, with no improvement in seizure frequency or severity, consistent with a pharmaco-resistant epilepsy profile.

Initiation of PN (20 mg/day) led to immediate seizure cessation and EEG normalization, with sustained seizure freedom thereafter. Based on these clinical findings, a PDE diagnosis was established despite negative results from *ALDH7A1* screening. At seven years old, after a completely seizure-free period and discontinuation of PN therapy, he experienced a seizure recurrence characterized by flexion spasms of the upper limbs (approximately 30 episodes per day) and neck hypotonia with loss of consciousness, prompting the reintroduction of PN treatment. This critical episode while awake was marked by buccal rhyme deviation and generalized clonus with loss of consciousness, lasting 2 min with a 20 min postictal phase, and required hospital admission. During hospitalization, a second critical episode was observed, featuring neck hypertonia with head and eyeball deviation to the left. He was treated with intravenous midazolam, which was ineffective, and PN (at 20 mg/kg/day) was reintroduced, with gradual dose escalation to 300 mg three times daily, leading to sustained seizure freedom and stable neurological status. The EEG during sleep, performed after reintroducing PN, showed no apparent paroxysmal abnormalities in a poorly structured baseline. A comprehensive neuropsychological assessment at age 13, using the Wechsler Intelligence Scale for Children—4th Edition (WISC-IV), revealed a disharmonic profile. His Verbal Comprehension Index was 134, Perceptual Reasoning Index was 87, and a Full-Scale IQ of 98. He could hold simple conversations and showed low self-esteem. An informal evaluation of academic skills indicated age-appropriate abilities. The proband underwent genetic testing after informed consent was obtained.

### 2.2. SNVs Analysis Using Trio-WES and SANGER Sequencing

Genomic DNA was extracted from the proband and the parents’ blood using a standard protocol. To explore potential constitutional single-nucleotide variants (SNVs) associated with the proband’s PDE, the proband’s DNA underwent PCR amplification and Sanger sequencing of the coding and adjacent intronic regions of the *PNPO* (NM_018129) and *ALDH7A1* (NM_001182.4) genes. Whole-exome sequencing (WES) and analysis of splicing consensus sequences (± 15 base pairs of neighboring intronic regions) were also performed.

### 2.3. Optical Genome Mapping (OGM) in the Trio

Ultra-high molecular weight gDNA (>150 kb) was extracted from the peripheral blood (EDTA) of the proband and his parents using the SP Blood & Cell Culture DNA Isolation Kit (Bionano Genomics, San Diego, CA, USA). The gDNA was labeled following the manufacturer’s instructions with the Bionano Prep Direct Label and Stain (DLS) protocol, and the data were analyzed on a Saphyr instrument (Bionano Genomics). A minimum of 500 Gb of data was collected. Bionano Solve v3.8.2 was used for de novo genome assembly at 80× coverage, trio analysis, variant calling, and annotation with default settings. Annotated variants were filtered for rare events (≤1% in the OGM control database), as described previously [6].

### 2.4. Pair-End Whole-Genome (PE-WGS) and Breakpoint Junctions Analysis

Genomic DNA was extracted from the proband’s blood with standard procedures and sequenced using an Illumina HiSeq 2000 platform with a 30 × PCR-free PE-WGS protocol (Illumina, San Diego, CA, USA). Reads were mapped to the human reference genome GRCh38/hg38 using BWA [7]. Structural variants (SVs) were called using Lumpy [8] and Delly [9]. They were then visualized and manually checked in the Integrative Genomics Viewer (IGV) genome browser to identify sample-specific SVs. All breakpoint junctions identified by OGM were validated through visual inspection of discordant paired-end reads and soft-clipped reads using the IGV genome browser.

### 2.5. Real-Time PCR (RT-PCR)

Two specific chromosome 1 target sequences for Real-Time PCR analysis (RT1 to RT2) were selected within non-repeated regions of the chromosome using Primer Express 3.0 software (Applied Biosystems, Foster City, CA, USA); a control amplicon was chosen with the same parameters in 1p22.2 (RT3). The primer sequences are listed in Table S1. We carried out amplification and detection on the Applied Biosystems QuantStudio 3 Real-Time PCR System using SYBR Green PCR Master Mix (Applied Biosystems).

### 2.6. Parental Origin Analysis of the 18q Deletion

We genotyped the family using a trio CGH-SNP array (180 k, Agilent, Santa Clara, CA, USA) (see Table S2).

### 2.7. Phenotypic and Genomic Assessment of T(1;18) Outcome

To evaluate sequence features and the potential impact of gene disruption on patient phenotype, we gathered the probability of loss-of-function intolerance (pLI) and LoF observed/expected upper bound fraction (LOEUF) from GnomAD v4.1.0, where pLI ≥ 0.9 and LOEUF < 0.35 indicate genes that are highly intolerant to loss of function. We also examined Topologically Associated Domains (TADs) that might be disrupted by chromothripsis breakpoints in lymphoblastoid cells (GM12878) [10] and neurogenic precursor cells (H1-NPC) [11] using the web-based 3D Genome Browser 2.0 (https://3dgenome.fsm.northwestern.edu/ (accessed on 1 September 2025)).

## 3. Result

**Figure 1:**
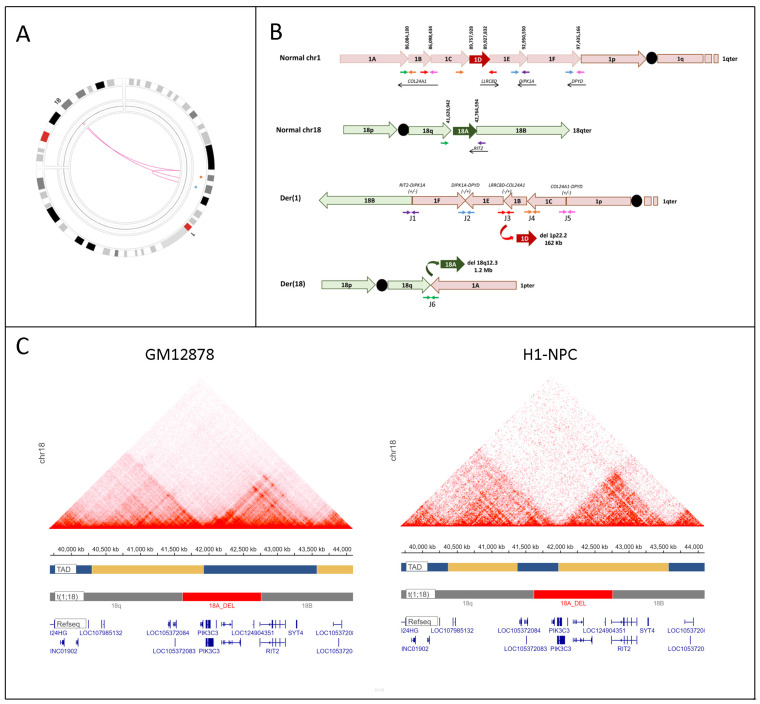
Characterization and interpretation of the complex t(1;18)(p22.3;q12.3). (**A**) OGM Circos plot: the central magenta lines connecting chromosomes 1 and 18 indicate the presence of a complex translocation. (**B**) Schematic representation of the rearrangement. The normal chromosome (chr) 1 is depicted in red, and the normal chromosome 18 is depicted in green. The two derivative chromosomes involved in the rearrangement [( der(1) and der(18)] show a translocated fragment (1A) transposed on the q-arm of der(18), a fragment (18B) translocated onto the p-arm of the der(1), and fragments (1F, 1Einv, 1Binv,1Cinv) reshuffled into the p-arm of der(1). The deleted fragments from chr18 and chr1 are depicted in dark green and red, respectively. Colored arrows at the bottom of chr1 and chr18 indicate discordant reads when aligned to the reference genome (hg38). The black arrow and +/− show transcription orientation of truncated protein-coding genes. The junctions (J) between the transposed fragments are numbered from 1 to 6 (J1–6; see Table S4). (**C**) Schematic illustration of the Topologically Associated Domain (TAD) structure (horizontal blue and yellow bars) encompassing the deleted fragment 18A (indicated with a horizontal red bar), removing the TAD boundary, as created by the 3D Genome Browser 2.0. The GM12878 Hi-C maps (left) [10] and H1-NPC (right) [11] are shown for each schematic view of TADs.

WES and Sanger sequencing analysis did not identify pathogenic or likely pathogenic SNVs in any PDE-associated genes. The trio-OGM analysis uncovered a de novo reciprocal unbalanced translocation t(1;18)(p22.3;q12.3) (Figure 1) and a recurrent duplication at 16p11.2 (BP4-5), inherited from the father (Figure S1). Combined with PE-WGS, OGM revealed that the two chromosomes involved in the translocation were shattered into ten fragments longer than 14.3 Kb, of which seven originated from chromosome 1p and three from chromosome 18q (Figure 1, Table S3). Two fragments were lost, covering 1.1 Mb at 18q12.3 (fragment 18A) and 169 kb at 1p22.2 (fragment 1D), the latter identified by PE-WGS analysis and confirmed using RT-PCR (Figure S2). The 18q12.3 deletion on the paternal chr18 (Table S2) removed *PIK3C3* (MIM:602609) and the last exon of *RIT2* (MIM:609592), while the 1p22.2 deletion affected the first two exons of *LRRC8D*. Four of the seven fragments from chromosome 1 (1C, 1B, 1E, 1F in Figure 1) were reshuffled into the short arm of the derivative chromosome 1 in a random order, with three of them inverted (1B, 1C, 1E), disrupting three protein-coding RefSeq genes (*COL24A1*, *DIPK1A*, *DPYD*) (Table S3). None of the breakpoint junctions resulted in potential fusion gene formation (Table S4). The presence of multiple clustered breakpoints creating five cis-junctions (J1–J5), a deletion at one junction (J3), and the pattern of the breakpoints—which includes microhomology regions ranging from 1 to 4 base pairs (Figures S3–S8)—consistent with repair-based mechanisms, along with the paternal origin of the rearrangement, collectively support classifying this complex translocation as chromothripsis [12].

## 4. Discussion

We describe a patient with normal intellectual development and PDE, evidenced by epileptic seizures starting at 5 months of age that were resistant to common antiepileptic drugs but responded to PN. Seizures recurred after PN discontinuation at age 7 years and were again managed with PN, leading to a significant reduction in seizure frequency and severity until age 13 years. Because WES did not identify clinically relevant variants in known PN metabolism genes, we performed OGM to investigate potential SVs underlying his condition. OGM revealed a complex de novo unbalanced t(1;18)(p22.3;q12.3) (Figure 1) and a recurrent duplication of BP4-5 16p11.2 inherited from his unaffected father (Figure S1).

The t(1;18) is classified as chromothripsis, an event characterized by the random shattering and reshuffling of clustered chromosome regions within a single catastrophic event [10]. After confirming the WES results, PE-WGS identified additional genes affected by the rearrangement, specifically *RIT2*, *PIK3C3*, *COL24A1*, *LRRC8D*, *DIPK1A*, *DPYD*, and *LRRC8D* (Table S3). None of which is an established epilepsy gene. Among these, only *DPYD* appears in the OMIM Morbid Map; however, DPYD-related disease (MIM #274270) is an autosomal recessive disorder of pyrimidine catabolism, making monoallelic disruption an unlikely explanation for the patient’s phenotype. Gene-constraint metrics also argue against haploinsufficiency for *COL24A1* and *DIPK1A* (tolerant to loss-of-function in gnomAD v4.1.0). Only *LRRC8D* is intolerant to LoF variants (Pli = 0.98, o/e = 0.35), although its known biological role relates to cellular drug uptake rather than a specific neurological phenotype [13].

Although RIT2 shows good tolerance to loss-of-function variants (pLI = 0; gnomAD v4.1.0), it is a gene of interest because it is exclusively expressed in the brain (GTEx, V6 release) and encodes a neuronal-specific guanosine triphosphatase essential for neuronal development and function [14]. Additionally, deletions overlapping with the 18q12.3 deletion in our patient—which, aside from *RIT2* disruption, lead to the loss of one copy of *PIK3C3*—have been documented in several other patients, summarized in Bouquillon et al., 2010 [15], who share with our patient a history of seizures, abnormal EEG, and limited speech. Moreover, among the genes within a short region of overlap of these deletions, the *SYT4* gene (MIM 600103)—which encodes a highly conserved membrane protein involved in synaptic function—may play a role in proximal 18q deletion syndromes as a factor predisposing to epilepsy [15,16]. Although *SYT4* was not included in our patient’s 18q12.3 deletion, the deleted fragment 18A (Figure 1C) encompasses a TAD boundary, in both lymphoblastoid [10] and neurogenic precursor [11] cells, leading to the fusion of two adjacent TADs, one of which includes *SYT4.* Structural variations that disrupt the 3D genome are known to cause diseases, including developmental disorders [17]. Therefore, it seems likely that the rearrangement of higher-order chromatin structures as a result of the 18q12.3 deletion or the t(1;8) conformation itself may have contributed to our patient’s clinical presentation.

The recurrent 16p11.2 BP4-5 duplication is associated with variable neurodevelopmental outcomes, including an increased seizure risk [MIM #614671], although the penetrance is incomplete. The duplication is paternally inherited from an unaffected carrier in this family.

Reported individuals with 16p11.2 BP4-5 duplications, either de novo or inherited from an unaffected parent, exhibit various seizure types and differing responses to standard AEDs, yet none were documented to have a clear pyridoxine dependence [18,19,20]

Extensive evidence shows that incompletely penetrant, variably expressive CNVs, including the 16p11.2 BP4-5 duplication, can be explained by a second diagnosis or an additional variant of uncertain significance, such as another CNV or single-gene variant, which could potentially influence the expressivity of 16p11.2 rearrangements [21,22].

Thus, we can not rule out that the final phenotype in our patient is influenced by currently uncharacterized dysregulatory effects resulting from an interplay between the chromothriptic t(1;18) and the proximal 16p11.2 duplication.

Although the molecular data do not implicate a single gene or locus as causal for seizure in this individual, a critical aspect of our patient’s phenotype is the true PN *dependence* rather than mere PN *responsiveness*. This distinction, though often blurred in clinical practice, carries significant diagnostic and therapeutic implications [2]. Several key features in our case definitively establish PN dependence: (1) complete pharmacoresistance to multiple conventional AEDs (phenobarbital, levetiracetam, valproic acid) despite adequate dosing and therapeutic levels; (2) dramatic and reproducible seizure cessation within hours of PN administration; (3) immediate seizure recurrence upon PN withdrawal at age seven, despite a prolonged seizure-free period; and (4) sustained seizure freedom only with continuous PN therapy, requiring escalation to 300 mg three times daily.

This pattern of absolute treatment dependence contrasts sharply with pyridoxine-responsive epilepsy, where seizures may show partial improvement with pyridoxine supplementation but can be adequately controlled with standard AEDs [23,24]. In pyridoxine-responsive cases, its role is adjunctive rather than indispensable for achieving seizure control. The pharmaco-resistance profile observed in our patient—characterized by complete failure of multiple first-line and second-line AEDs—is a hallmark of metabolic epilepsies, including classical PDE [25]. This refractory nature prior to PN introduction strongly suggests an underlying metabolic disturbance affecting PN-dependent pathways, despite the absence of mutations in canonical PDE genes.

Furthermore, the requirement for dose escalation to high PN levels (300 mg TID, equivalent to approximately 20–25 mg/kg/day for a 13-year-old) approaches therapeutic ranges used in confirmed PDE cases. This dose-dependency, coupled with the complete seizure freedom achieved only at these higher doses, provides additional evidence that PN is not merely modulating seizure threshold but is compensating for a fundamental metabolic requirement.

The normal intellectual development observed in our patient, despite true PN dependence, represents an unusual but increasingly recognized phenotypic variant.

Classical PDE caused by *ALDH7A1* variants frequently results in intellectual disability due to accumulation of neurotoxic intermediates (α-aminoadipic semialdehyde and piperideine-6-carboxylate) even when seizures are controlled [26]. The preservation of cognition in our case may suggest: (1) early and consistent PN treatment preventing significant neurotoxic accumulation; (2) involvement of alternative metabolic pathways with different neurotoxic profiles; or (3) protective genetic modifiers within the complex genomic background that mitigate neurotoxicity. This presentation with preserved cognition has been documented in small cohorts of PDE patients, particularly those diagnosed and treated early [3,4], and may represent a distinct phenotypic subgroup worthy of further genetic investigation.

## 5. Conclusions

OGM played a crucial role in identifying the genetic complexity associated with both the chromothriptic t(1;18) and the 16p11.2 BP4-5 duplication, which may create a unique metabolic environment where pyridoxine-dependent pathways are dysregulated through non-canonical mechanisms. Therefore, we propose that our patient exemplifies a paradigm of non-canonical pyridoxine-dependent epilepsy—a condition exhibiting the clinical hallmarks of true PDE (pharmacoresistance, dramatic PN response, dependence for seizure control) without mutations in known PN metabolism genes. This challenges the traditional gene-centric definition of PDE and suggests that structural genomic variations affecting neuronal function and metabolism can phenocopy classical PDE through alternative pathways. Recognizing this broader phenotypic spectrum has important clinical implications: empirical PN trials should be considered in all cases of early-onset pharmaco-resistant epilepsy, regardless of routine genetic testing results, as delayed diagnosis can lead to ongoing seizures, developmental regression, and unnecessary exposure to ineffective AEDs. The preservation of cognitive function, uncommon in typical PDE, may reflect a milder phenotype, possibly influenced by a different genetic background that limits the accumulation of neurotoxic metabolites. This case expands the phenotypic and genetic spectrum of pyridoxine-dependent epilepsies and underscores the importance of maintaining a broad differential diagnosis in pharmacoresistant infantile seizures.

## Data Availability

The original contributions presented in this study are included in the article. Further inquiries can be directed to the corresponding author.

## Supplementary Materials

The following supporting information can be downloaded at: https://www.mdpi.com/article/10.3390/genes16111334/s1, Table S1: Primer’s sequences and genomic location, Table S2: Informative SNPs from the 180K CGH+SNP array platform (G4890A, Agilent Technologies) showing the paternal origin of the 18q12.3 deletion, Table S3: Chromosome fragments participating in the complex t(1;18)(p22.3;q12.3)dn, Table S4: Breakpoint junctions (J1–J6), Figure S1: Recurrent duplication 16p11.2 BP4-5, Figure S2: Real-time PCR results for the proband and his parents indicating de novo 1p22.2 deletion in the proband with probes RT1: del_1p22.2_chr1:89,821,692-89,821,759 (hg38); RT2: del_1p22.2bis: chr1:89,843,837-89,843,895 (hg38); RT3: Ctrl_chr1:89,934,630-89,934,690 ( hg38), Figure S3: IGV visualization of the breakpoint junction 1 (J1) between fragments 18 Binv_1F on the derivative (der) chromosome 1 (see also Figure 1 in the main text). Dashed vertical lines indicate breakpoints of fragments 18B (panel left) and 1F (panel right). Blast results of soft-clipped read sequences at J1 showed a microhomology of two bps (TC), Figure S4: IGV visualization of the breakpoint junction 2 (J2) between fragments 1F_1Einv on the derivative (der) chromosome 1 (see Figure 1 in the main text). Dashed vertical lines indicate breakpoints of each fragments 1F (panel left) and 1Einv (panel right). Blast results of soft-clipped read sequences at J2 showed a microhomology of 4 bps (TCTC), Figure S5: IGV visualization of the breakpoint junction 3 (J3) between fragments 1Einv_1Binv on the derivative (der) chromosome 1. Dashed vertical lines indicate breakpoints of each fragment 1Einv (panel right) and 1Binv (panel left). Blast results of soft-clipped read sequences at J3 were not informative due to repeated sequences (LINE, see Table S1), Figure S6: IGV visualization of the breakpoint junction 4 (J4) between fragments 1Binv_1Cinv on the derivative (der) chromosome 1. Dashed vertical lines indicate breakpoints of each fragment 1Binv (panel left) and 1Cinv (panel right). Blast results of soft-clipped read sequences at J4 showed a microhomology of 1 bp (G), Figure S7: IGV visualization of the breakpoint junction 5 (J5) between fragments 1Cinv_1p on the derivative (der) chromosome 1. Dashed vertical lines indicate breakpoints of each fragments 1Cinv (panel left) and 1p (panel right). Blast results of soft-clipped read sequences at J5 showed a microhomology of 1 bp (T), Figure S8: IGV visualization of the breakpoint junction 6 (J6) between fragments 18q_1Ainv on the derivative (der) chromosome 18. Dashed vertical lines indicate breakpoints of each fragment 18q (panel right) and 1Ainv (panel left). Blast results of soft-clipped read sequences at J6 were not informative.

## Abbreviations

The following abbreviations are used in this manuscript:

PDE	Pyridoxine-dependent epilepsy
PN	Pyridoxine
AED	Resistant to antiepileptic drugs
LD	Optical genome mapping
PE-WGS	Paired-end whole-genome sequencing
ID	Intellectual disability
SV	Structural variant
