# Supplementary material for: Chromothriptic Translocation t(1;18): A Paradigm of Genomic Complexity in a Child with Normal Intellectual Development and Pyridoxine-Dependent Epilepsy
Source: Genes (Basel). 2025 Nov 5;16(11):1334. doi: 10.3390/genes16111334 (PMC12652939; doi:10.3390/genes16111334)
Supplement: Supplementary file 1 [file genes-16-01334-s001.zip › genes-3969899-supplementary.pdf]

TITLE : Chromothriptic translocation t(1;18): a paradigm of genomic complexity in a child with normal intellectual development and pyridoxine-dependent epilepsy

SUPPLEMENTARY TABLES S1-S4

Table S1 Primer's sequences and genomic location

	primer seq (5'-3')	genomic location (hg38)
RT1	GTTTGGTGTCTGGCCTTGCT	chr1:89,821,692-89,821,759
	GGAGGCGATTCTGGAGAAGTT	
RT2	AGCACTGCTAGTGTCGGATCTG	chr1:89,843,837-89,843,895
	GGCGGGCAAGTGAAGCT	
RT3	GCCACTGCCCTGCAAAAG	chr1:89,934,630-89,934,690
	CATCTCAAGTGATCGCGAAGAA	

Table S2: Informative SNPs from the 180K CGH+SNP array platform (G4890A, Agilent Technologies) showing the paternal origin of the 18q12.3 deletion

SNP ID	Chromosome	Position map (hg38)	proband	father	mother	Parental origin
rs1433928	chr18	41,665,002	C	TT	CC	paternal
rs11082255	chr18	41,949,795	G	AA	GA	paternal

Table S3: Chromosome fragments participating in the complex t(1;18)(p22.3;q12.3)dn

Chromosome	Fragment	Start (hg38)	Stop (hg38)	Size	Interrupted genes (orientation)	Repeat
1	1A	1pter	86084100	86 Mb	*COL24A1 int-7* (NM_001349955.1) (-)	
	1B	86084100	86098434	14.3 Kb	*COL24A1 int-5* (NM_001349955.1) (-)	LINE (L1)
	1C	86098434	89757928	3.6 Mb	*LOC107985743*	
	1D_DEL	89757928	89927832	169.9 Kb	*LRRC8D int-2* (NM_001134479.2) (+)	
	1E	89927832	92950550	3 Mb	*DIPK1A int-1* (NM_001006605.5)(-)	
	1F	92950550	97435165	4.4 Mb	*DPYD int-14* (NM_000110.4)(-)	
	1p	97435167	cen	24.2 Mb		
18	18q	cen	41620942	20.1 Mb	*KC6 (lncRNA)* (-)	
	18A_DEL	41620942	42764594	1.1 Mb	*RIT2 int-4* (NM_002930.4) (-)	
	18B	42764594	18qter	37.6 Mb		

Legend: cen: centromere; DEL: deletion; int: intron; DEL: deletion

Table S4: Breakpoint junctions (J1-J6)

Derivative chr	Junction	Fragments	Genomic coordinates (hg38)	Feactures at Junction	Gene Fusion [orientation at rearrangment]	Repeats
der1	J1	18B inv_1F	chr18:42764594 _ chr1:92950549	2 bp microhomology (TC)	*RIT2(-)-DIPK1A(-) [+/-]*	
	J2	1F_1Einv	chr1:97435165 _ chr1:92950550	4 bp microhomology (TCTC)	*DPYD(-)-DIPK1A(-) [-/+]*	
	J3	1Einv_1Binv	chr1:89.927.832_ chr1:86.098.432 *	n.d. *	*LRRC8D(+)-COL24A1 (-) [-/+]*	none/LINE
	J4	1Binv_1Cinv	chr1:86084100_ chr1:89757928	1 bp microhomology (G)		
	J5	1Cinv_1p	chr1:86098438 _ chr1:97435167	1 bp microhomology (T)	*COL24A1 (-)-DPYD(-) [+/-]*	
der18	J6	18q_1Ainv	chr18:41620942_ chr1:86084103 *	n.d. *		

Legend: n.d*: Not determined due to lack of BLAT results available

SUPPLEMENTARY FIGURES S1-S8

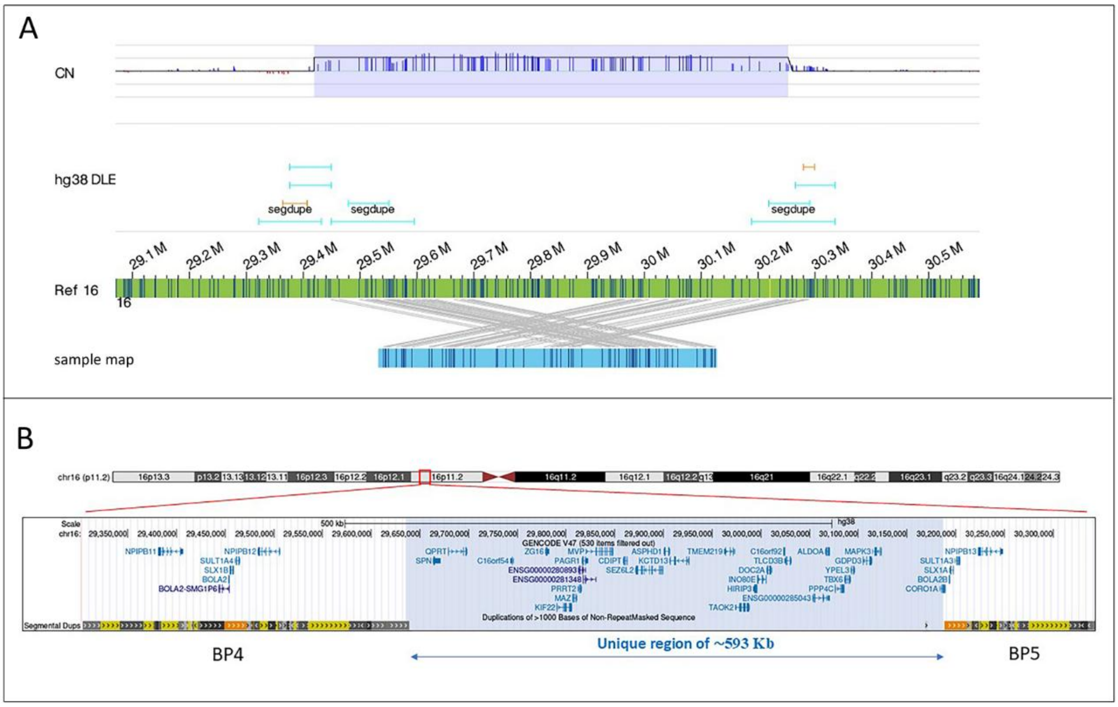

Figure S1: Recurrent duplication 16p11.2 BP4-5

(A) Visualization of the tandem 16p11.2 bordered by the blocks of segmental duplication. From top to bottom: copy number (CN) track showing the 16p11.2 duplication picked up by the copy number (CNV_call), segmental duplication track, and structural variant (SV_call) algoritms. The alignment between reference maps of chromosome 16 (Ref 16, green bar) and our patient's map (sample map, blue bar) represented as gray strings, indicate the presence of 16p11.2 tandem duplication

(B) Magnified view of the 16p11.2 BP4-BP5 duplicated region of ~593 kb, shaded blue. From top to bottom: UCSC genes (GRCh38/hg38); segmental duplication blocks colored based on the similarity degree, ranging from 90 to 99% according to the UCSC genome browser (https://genome.ucsc.edu/, GRCh38)

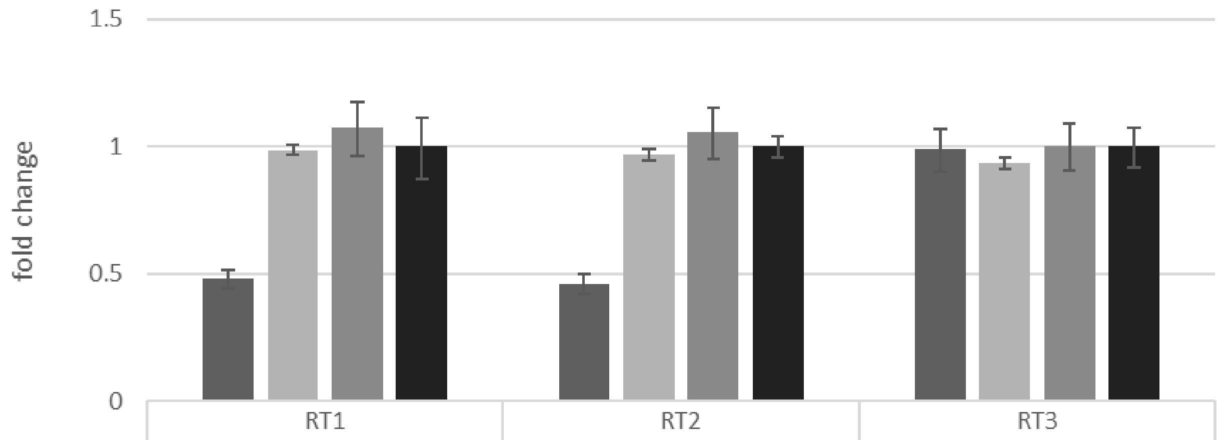

	RT1	RT2	RT3
Proband	0.481240279	0.459349021	0.987969372
Father	0.986904549	0.966342172	0.935029097
Mother	1.071828064	1.056596969	1.001755931
Ctrl	1	1	1

Figure S2: **Real-time PCR results** for the proband and his parents indicating *de novo* 1p22.2 deletion in the proband with probes RT1: del_1p22.2_chr1:89,821,692-89,821,759 (hg38); RT2: del_1p22.2bis: chr1:89,843,837-89,843,895 (hg38) ; RT3: Ctrl_chr1:89,934,630-89,934,690 (hg38)

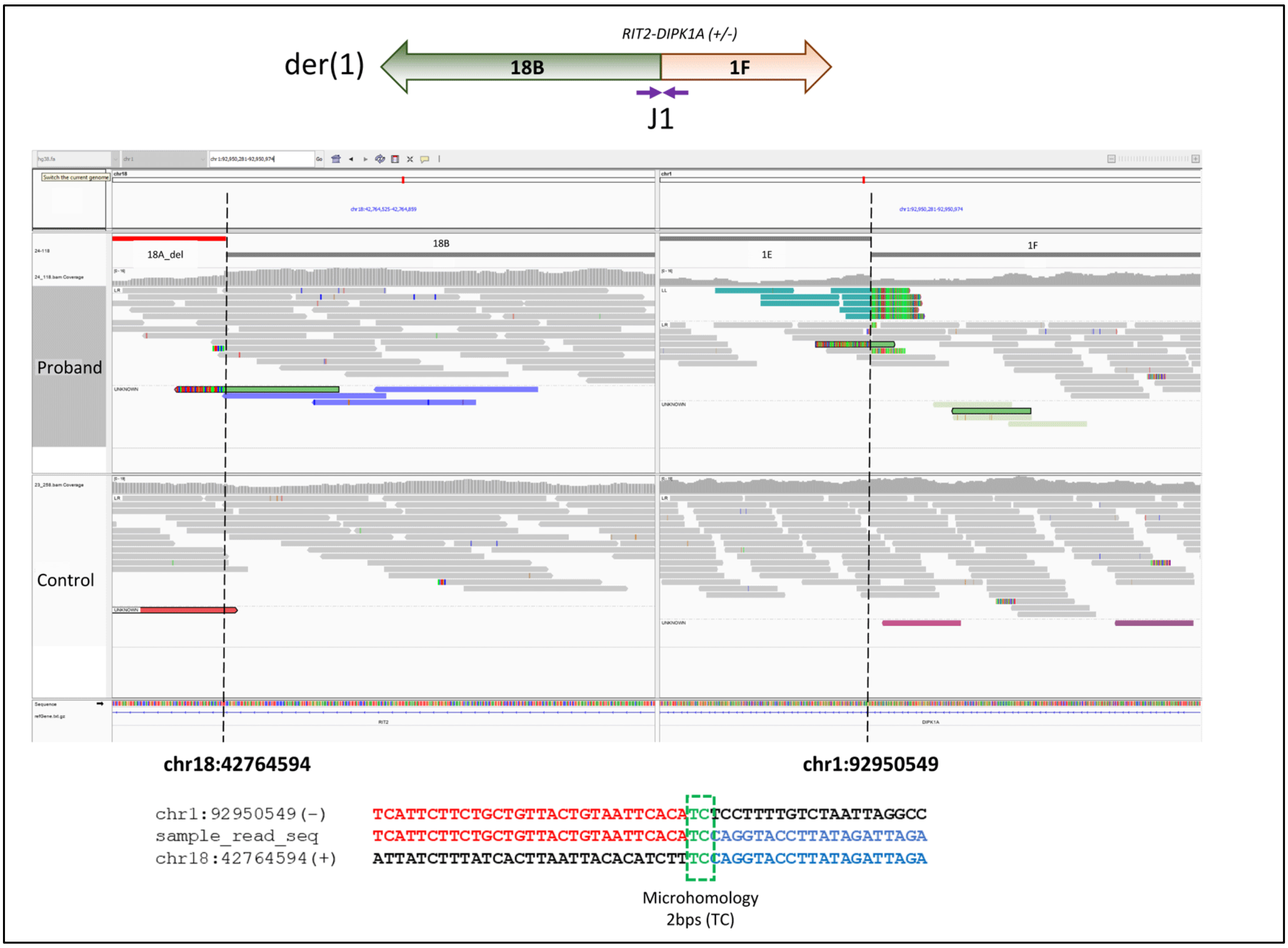

Figure S3: IGV visualization of the breakpoint junction 1 (J1) between fragments 18 Binv_1F on the derivative (der) chromosome 1 (see also Fig.1 in the main text). Dashed vertical lines indicate breakpoints of fragments 18B (panel left) and 1F (panel right). Blast results of soft-clipped read sequences at J1 showed a microhomology of two bps (TC)

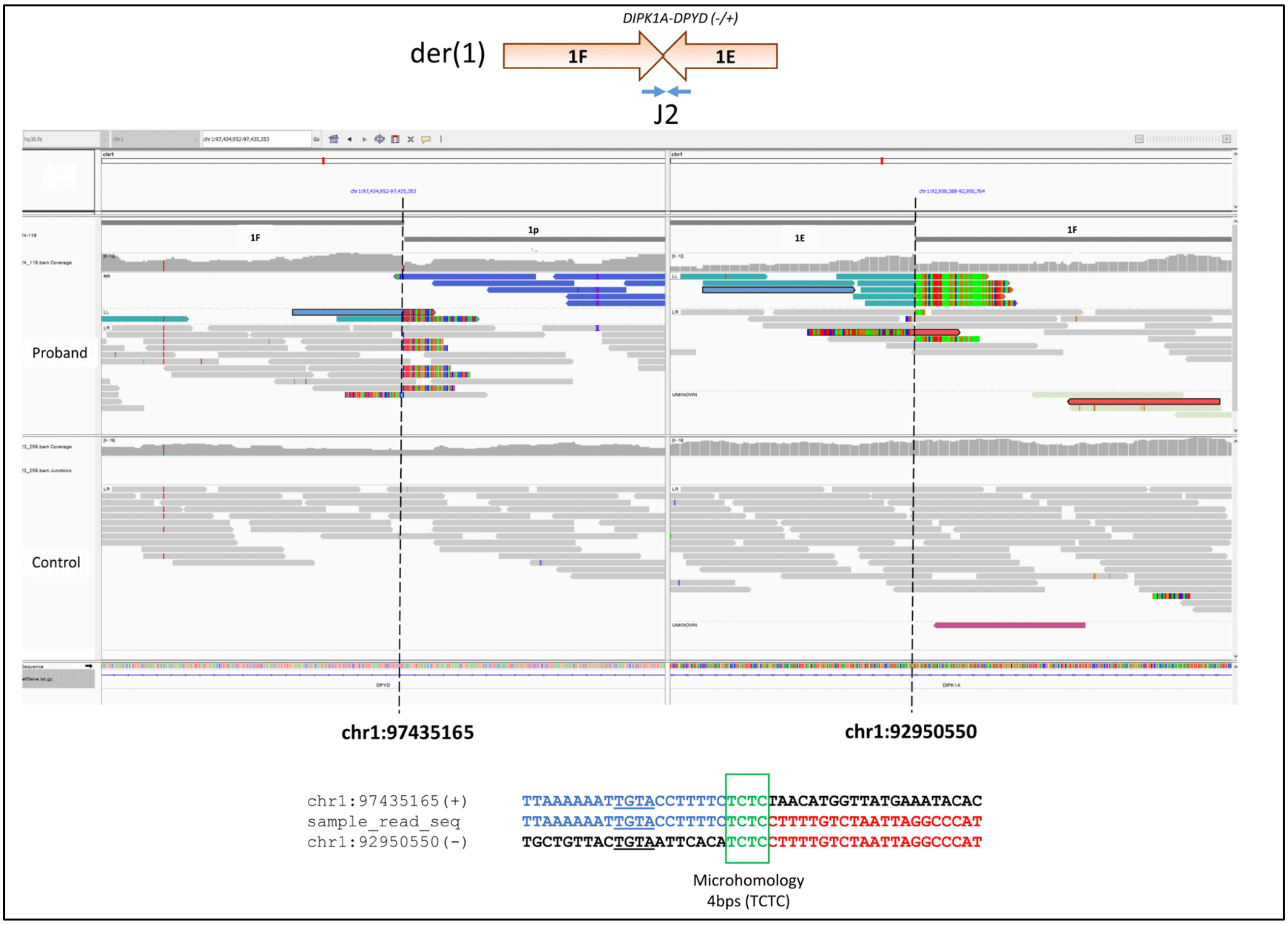

Figure S4: IGV visualization of the breakpoint junction 2 (J2) between fragments 1F_1Einv on the derivative (der) chromosome 1 (see Fig.1 in the main text). Dashed vertical lines indicate breakpoints of each fragments 1F (panel left) and 1Einv (panel right). Blast results of soft-clipped read sequences at J2 showed a microhomology of 4 bps (TCTC).

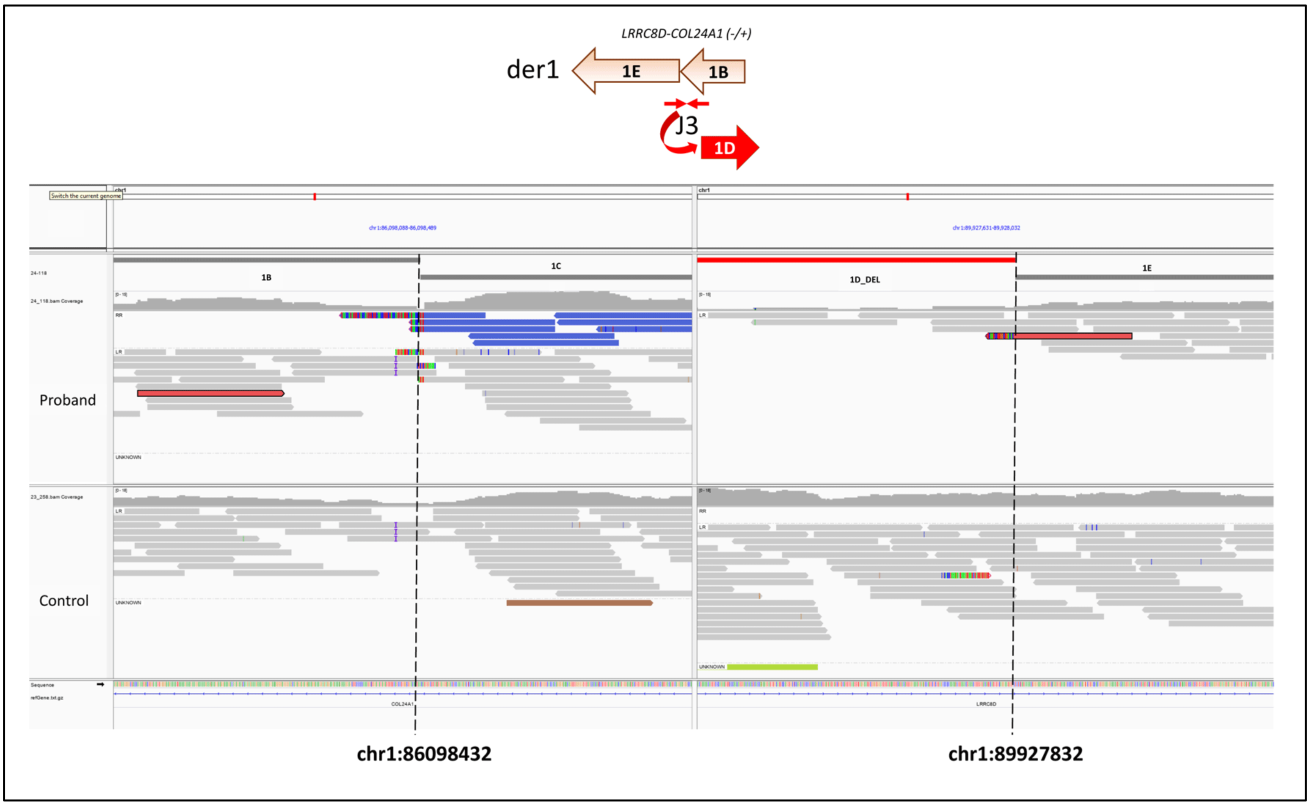

Figure S5: IGV visualization of the breakpoint junction 3 (J3) between fragments 1Einv_1Binv on the derivative (der) chromosome 1. Dashed vertical lines indicate breakpoints of each fragment 1Einv (panel right) and 1Binv (panel left). Blast results of soft-clipped read sequences at J3 were not informative due to repeated sequences (LINE, see Table S1).

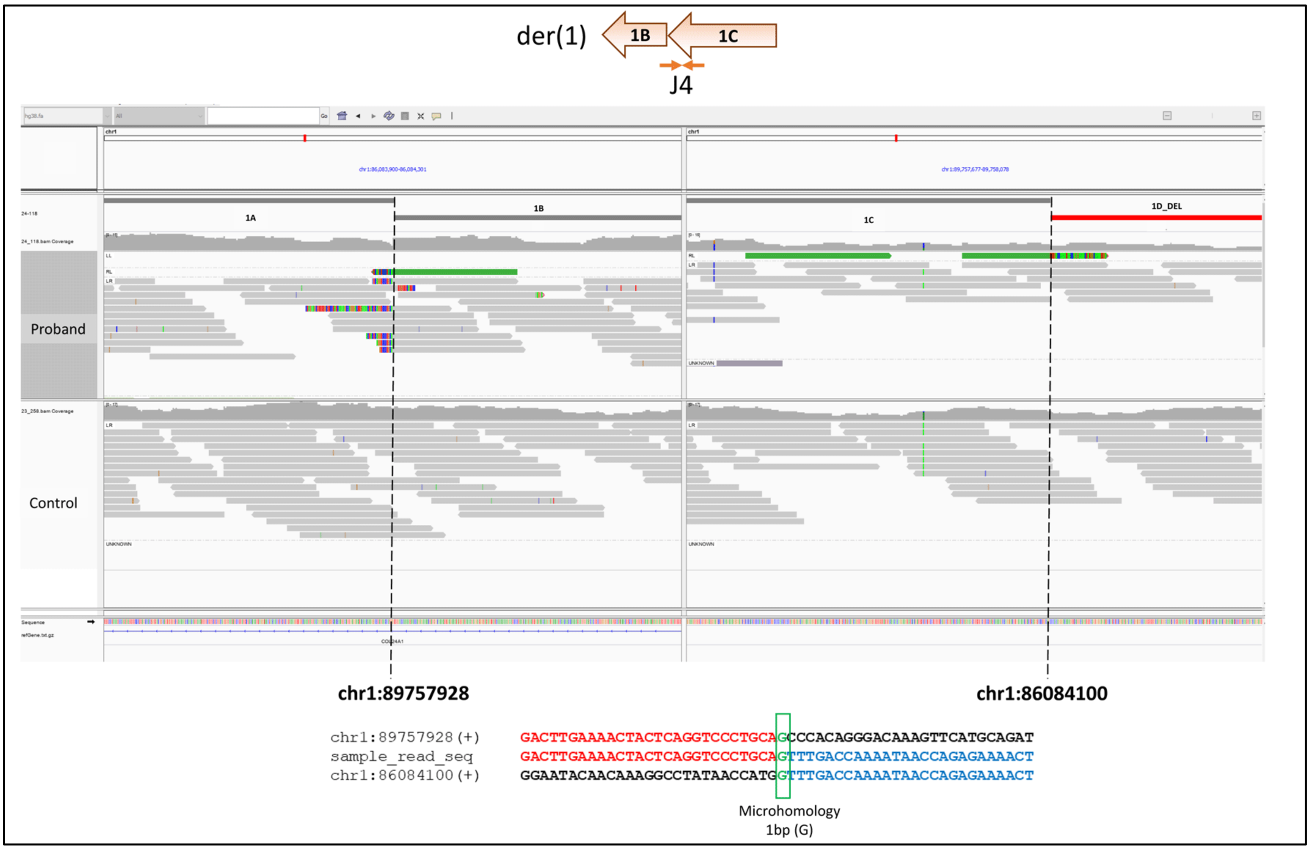

Figure S6: IGV visualization of the breakpoint junction 4 (J4) between fragments 1Binv_1Cinv on the derivative (der) chromosome 1. Dashed vertical lines indicate breakpoints of each fragment 1Binv (panel left) and 1Cinv (panel right). Blast results of soft-clipped read sequences at J4 showed a microhomology of 1 bp (G).

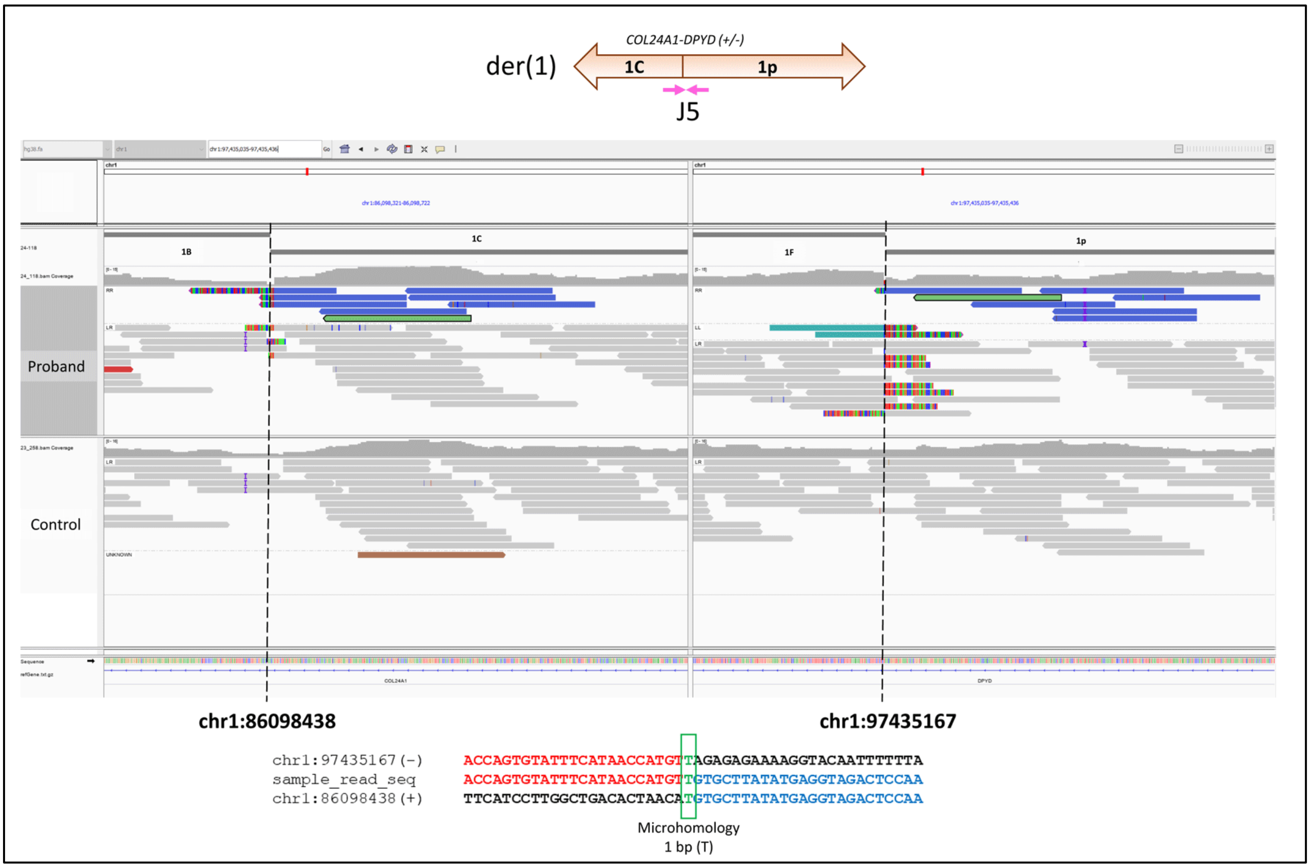

Figure S7: IGV visualization of the breakpoint junction 5 (J5) between fragments 1Cinv_1p on the derivative (der) chromosome 1 . Dashed vertical lines indicate breakpoints of each fragments 1Cinv (panel left) and 1p (panel right). Blast results of soft-clipped read sequences at J5 showed a microhomology of 1 bp (T).

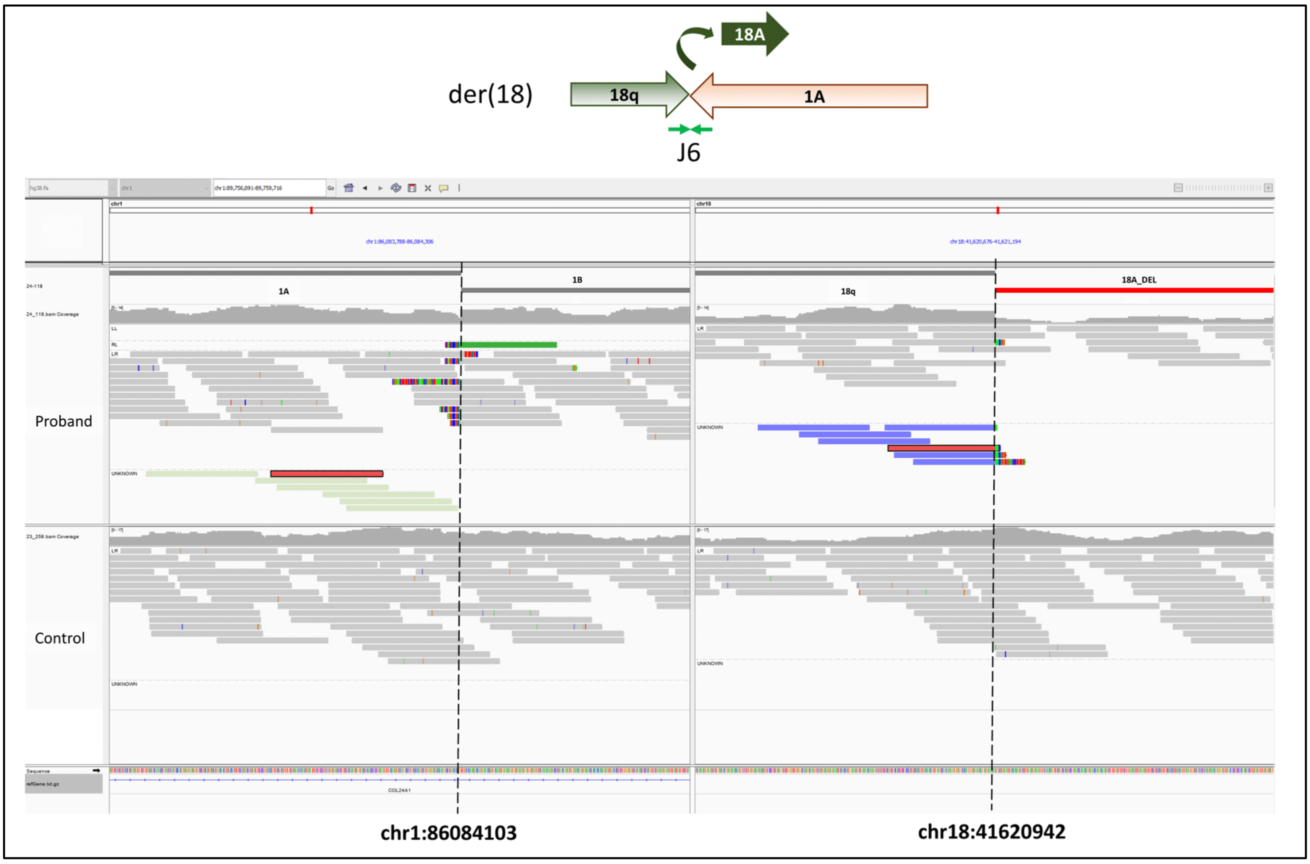

Figure S8: IGV visualization of the breakpoint junction 6 (J6) between fragments 18q_1Ainv on the derivative (der) chromosome 18. Dashed vertical lines indicate breakpoints of each fragment 18q (panel right) and 1Ainv (panel left). Blast results of soft-clipped read sequences at J6 were not informative.
